# Integrated radiogenomics models predict response to neoadjuvant chemotherapy in high grade serous ovarian cancer

**DOI:** 10.1038/s41467-023-41820-7

**Published:** 2023-10-24

**Authors:** Mireia Crispin-Ortuzar, Ramona Woitek, Marika A. V. Reinius, Elizabeth Moore, Lucian Beer, Vlad Bura, Leonardo Rundo, Cathal McCague, Stephan Ursprung, Lorena Escudero Sanchez, Paula Martin-Gonzalez, Florent Mouliere, Dineika Chandrananda, James Morris, Teodora Goranova, Anna M. Piskorz, Naveena Singh, Anju Sahdev, Roxana Pintican, Marta Zerunian, Nitzan Rosenfeld, Helen Addley, Mercedes Jimenez-Linan, Florian Markowetz, Evis Sala, James D. Brenton

**Affiliations:** 1 Department of Oncology, University of Cambridge, Cambridge, UK; 2 Cancer Research UK Cambridge Centre, University of Cambridge, Cambridge, UK; 3 Department of Radiology, University of Cambridge, Cambridge, UK; 4 Centre for Medical Image Analysis and AI (MIAAI), Danube Private University, Krems, Austria; 5 Cancer Research UK Cambridge Institute, University of Cambridge, Cambridge, UK; 6 Cambridge University Hospitals NHS Foundation Trust, Cambridge, UK; 7 Department of Biomedical Imaging and Image-guided Therapy, Medical University of Vienna, Vienna, Austria; 8 Department of Information and Electrical Engineering and Applied Mathematics, University of Salerno, Fisciano, SA Italy; 9 Department of Pathology, Amsterdam UMC location Vrije Universiteit Amsterdam, Amsterdam, The Netherlands; 10 Department of Cellular Pathology, Barts Health NHS Trust, London, UK; 11 Department of Radiology, Barts Health NHS Trust, London, UK; 12 “Iuliu Hatieganu” University of Medicine and Pharmacy, Cluj-Napoca, Romania; 13 Department of Radiology, County Clinical Emergency Hospital, Cluj-Napoca, Romania; 14 Department of Surgical and Medical Sciences and Translational Medicine, Sapienza University of Rome-Sant’Andrea University Hospital, Rome, Italy; 15 Dipartimento di Scienze Radiologiche ed Ematologiche, Universita Cattolica del Sacro Cuore, Rome, Italy; 16 Dipartimento Diagnostica per Immagini, Radioterapia Oncologica ed Ematologia, Policlinico Universitario A. Gemelli IRCCS, Rome, Italy; 17 Western Balkans University, Tirana, Albania

**Keywords:** Ovarian cancer, Predictive markers, Cancer imaging

## Abstract

High grade serous ovarian carcinoma (HGSOC) is a highly heterogeneous disease that typically presents at an advanced, metastatic state. The multi-scale complexity of HGSOC is a major obstacle to predicting response to neoadjuvant chemotherapy (NACT) and understanding critical determinants of response. Here we present a framework to predict the response of HGSOC patients to NACT integrating baseline clinical, blood-based, and radiomic biomarkers extracted from all primary and metastatic lesions. We use an ensemble machine learning model trained to predict the change in total disease volume using data obtained at diagnosis (*n* = 72). The model is validated in an internal hold-out cohort (*n* = 20) and an independent external patient cohort (*n* = 42). In the external cohort the integrated radiomics model reduces the prediction error by 8% with respect to the clinical model, achieving an AUC of 0.78 for RECIST 1.1 classification compared to 0.47 for the clinical model. Our results emphasize the value of including radiomics data in integrative models of treatment response and provide methods for developing new biomarker-based clinical trials of NACT in HGSOC.

## Abstract (web summary)

Response to treatment in high grade serous ovarian carcinoma (HGSOC) is highly variable. Here, the authors leverage a radiogenomic model to predict neoadjuvant chemotherapy response in HGSOC, including clinical data, medical imaging, and blood-based biomarkers such as CA-125 and ctDNA features.

## Introduction

High-grade serous ovarian carcinoma (HGSOC) is a major therapeutic challenge as it typically presents with advanced, multi-site metastatic disease. Neoadjuvant chemotherapy (NACT) followed by delayed primary surgery (DPS) is now the most frequent treatment strategy for advanced HGSOC^1,2^. However, response is variable, and 39% of patients do not obtain any objective benefit from neoadjuvant carboplatin and paclitaxel^3–5^. Variability in response is partly driven by the complexity of HGSOC, which spans a large range of scales—from macroscopic metastatic tumour volumes observed on radiological imaging to microscopic tumour-immune microenvironments and sub-microscopic genomic diversity^6–8^. Patient care would be substantially improved if different sub-populations could be identified before treatment is started, for example by identifying likely non-responders who could receive immediate primary surgery.

So far, predictive studies have focused on individual data streams, such as clinical features^9,10^, CA-125^11–13^, computed tomography (CT) imaging^14,15^, and circulating tumour DNA (ctDNA)^16^. The superior predictive power of integrative models for complex endpoints is well demonstrated in several cancer types^17–19^, but training such models requires large, well-annotated, multi-omic datasets, which have not been available for NACT treatment of HGSOC. In particular, radiological imaging is the only data source that non-invasively captures the spatial heterogeneity of metastatic disease, but has so far been underused. Existing radiomics studies focus only on correlations^14,15^, rather than combined predictive power, and do not consider NACT.

Here, we present *IRON*, an integrative radiogenomic framework to predict the volumetric response of heterogeneous, multi-site ovarian cancer to NACT. We used two independent, highly annotated data sets including clinical, chemotherapy treatment, CA-125, ctDNA, and radiomics features extracted from all primary and metastatic disease at diagnosis. The features were used as an input to an ensemble machine learning model trained to predict volume shrinkage during NACT. We validated the approach on an external training data set and demonstrated that radiomics features are essential to obtain significant predictive power. Our method is easily generalised and is applicable to other cancers with heterogeneous, multi-site disease.

## Results

**Fig. 1:**
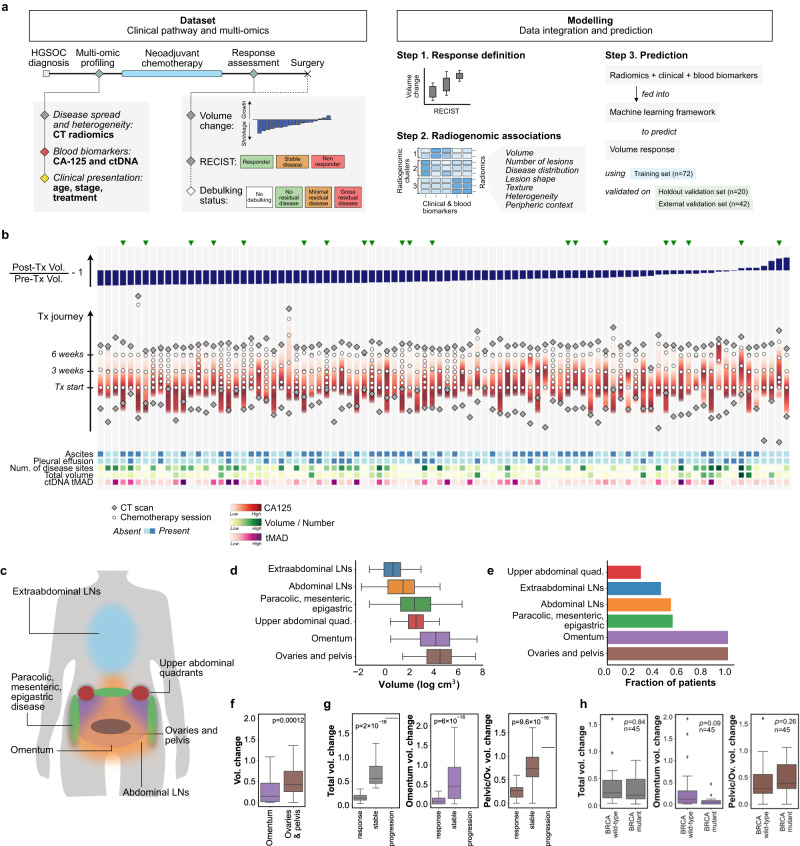
Structure of the study and main characteristics of the training cohort. **a** Key time points and variables in the dataset (left) and steps of the modelling strategy (right). See also Supplementary Fig. S1 for additional information. **b** Treatment courses of all 92 patients in the NeOV cohort, ordered by decreasing volumetric tumour response following NACT. Patients analysed in the hold-out validation set were randomly selected and are indicated with a green triangle. Treatment journeys progress vertically (bottom to top) and are aligned at the time of the first chemotherapy cycle. Additional biomarkers obtained at baseline are depicted in the bottom heatmap. **c** Sites of primary and metastatic disease in HGSOC. **d** Distribution of tumour volumes by site for patients in the training cohort. **e** Distribution of tumour sites by patient. **f** Volume changes of the omental and pelvic/ovarian disease for all patients in the training cohort. *p* value obtained from the two-sided Mann-Whitney *U* test. **g** Total and site-specific volume change stratified by RECIST 1.1 response status for the training cohort. *p* value obtained from the point biserial correlation coefficient, two-tailed. **h** Total and site-specific volume change stratified by *BRCA* mutation status. These figures are restricted to the *n* = 45 patients in the training cohort for whom the *BRCA* mutation status was known. *p* value obtained from the two-sided Mann-Whitney *U* test. Boxes indicate the upper and lower quartiles, with a line at the median. Outliers are shown as circles and identified via the interquantile range rule. Source data are provided as a Source Data file.

This study is based on data from two prospective observational studies from Addenbrooke’s Hospital (‘NeOv’, *n* = 92) and the Barts Health NHS Trust (‘Barts’, *n* = 42), respectively (Fig. 1a, Supplementary Fig. S1). Patients show variability in treatment regimes and response patterns (Fig. 1b). The NeOv dataset was randomly divided into a training set (*n* = 72) and a hold-out internal validation set (*n* = 20, Supplementary Fig. S1a). The training data set was used for the exploratory analyses and to train the machine learning models. The hold-out set was only used for performance assessment after all predictive models had been fully trained. The Barts data were used as an independent, external validation data set.

### Response patterns to NACT are heterogeneous

All primary and metastatic lesions identified on pre- and post-NACT CT scans were segmented and labelled (Fig. 1c). As expected, tumour in the omentum and pelvic/ovarian locations accounted for the majority of the disease burden at baseline and were the most frequent tumour locations (Fig. 1d, e). There were marked differences in response at the same anatomic sites between patients (Supplementary Fig. S3). Omental disease showed significantly better response than pelvic disease (Fig. 1f, g, h). Despite anatomic differences in response, use of response evaluation criteria in solid tumours (RECIST 1.1) was strongly correlated with total volume change (Fig. 1f). The volume of omental disease at baseline was higher (*p* = 0.05) in responders assessed by RECIST (complete or partial response; median = 85 cm^3^) compared to non-responders (stable disease or progression; median = 31 cm^3^).

While the number of disease locations at baseline was significantly correlated with response, there was no correlation with disease volume at baseline, either overall or in specific anatomic locations (Table 1). Taken together, these detailed volumetric data indicate that multivariable predictors are required to predict response to NACT rather than simple knowledge about disease burden and its anatomic distribution.

**Table 1:** Spearman correlation coefficients (*r*_*S*_) and corresponding two-sided *p* values (*p*) between baseline measurements of tumour burden and different assessments of treatment response

		Pre-chemotherapy									
		*TP53* MAF	CA-125	Oment. vol.	Pelvis/ovaries vol.	LN vol.	Total vol.	Number lesions	Summed diameter	Ascites	Pleural effusion
Volume change	*r*_*S*_	−0.05	−0.30	−0.07	0.12	−0.13	0.01	−**0.38**	−0.08	−0.15	−0.03
	*p*	0.93	0.07	0.90	0.62	0.60	0.96	**0.02**	0.90	0.55	0.93
Summed diam. change	*r*_*S*_	−0.06	−0.28	0.04	0.24	−0.16	0.15	−**0.34**	0.02	−0.03	−0.01
	*p*	0.93	0.08	0.93	0.16	0.55	0.55	**0.04**	0.93	0.93	0.96

*p* values are adjusted for multiple correction.

Significant correlations (*p* < 0.05) are highlighted in bold.

### ctDNA and CA-125 correlate with different types of disease burden

For all patients in the training and hold-out validation cohorts, ctDNA was assessed at baseline. We compared *TP53* mutant allele fraction (MAF), trimmed median absolute deviation from copy number neutrality based on shallow whole genome sequencing (t-MAD) and IchorCNA^20–23^. In addition, we explored the use of computed haploid genome equivalents per millilitre (hGE/ml)^24^. All measures were highly correlated (*p* < 0.0001, Supplementary Fig. S1c) and therefore, *TP53* MAF was included in univariable analyses.

Total disease burden at baseline (total volume, number of lesions, and summed RECIST 1.1 diameters) correlated significantly with both CA-125 and *TP53* MAF (Table 2). Neither baseline CA-125 nor *TP53* MAF correlated significantly with response, but did show a significant positive correlation with the summed RECIST 1.1 diameters post-chemotherapy (Table 1).

**Table 2:** Spearman correlation coefficients (*r*_*S*_) and corresponding two-sided *p* values (*p*) for pre- and post-NACT measurements of tumour burden versus blood biomarkers measured at baseline

		Pre-chemotherapy							
		Oment. vol.	Pelvis/ovaries vol.	LN vol.	Total vol.	Number lesions	Summed diameter	Ascites	Pleural effusion
Baseline *TP53* MAF	*r*_*S*_	0.04	**0.37**	0.26	**0.37**	**0.32**	**0.48**	0.05	0.13
	*p*	0.77	**0.005**	0.06	**0.005**	**0.02**	**0.0003**	0.77	0.36
Baseline CA-125	*r*_*S*_	**0.41**	**0.38**	0.21	**0.48**	**0.28**	**0.45**	**0.28**	0.23
	*p*	**0.002**	**0.005**	0.13	**0.0003**	**0.04**	**0.0009**	**0.04**	0.10

		Post-chemotherapy							
		Oment. vol.	Pelvis/ovaries vol.	LN vol.	Total vol.	Number lesions	Summed diameter	Ascites	Pleural effusion
Baseline *TP53* MAF	*r*_*S*_	−0.04	**0.30**	0.13	**0.29**	0.19	**0.41**	0.03	0.13
	*p*	0.79	**0.03**	0.36	**0.03**	0.17	**0.003**	0.79	0.36
Baseline CA-125	*r*_*S*_	0.10	0.23	0.10	0.22	0.20	**0.32**	−0.07	−0.16
	*p*	0.50	0.10	0.50	0.11	0.15	**0.02**	0.63	0.24

*p* values are adjusted for multiple correction.

Significant correlations (*p* < 0.05) are highlighted in bold.

Baseline CA-125 correlated with omental disease and pelvic/ovarian disease volume measured before chemotherapy. Similarly, baseline *TP53* MAF correlated significantly with pelvic/ovarian disease volume measured both before and after chemotherapy. However, ctDNA measurements did not correlate with omental disease at either time point (Table 2). This suggests that high *TP53* MAF at baseline could be a specific indicator for high disease burden in the ovaries or pelvis, which tends to show poorer response (Fig. 1f).

### Radiomics features correlate with clinical and biological characteristics

To capture the radiological complexity of the disease we defined several collections of radiomics features (Supplementary Data 1). Volumes and number of lesions were calculated for each of the relevant anatomical sites, in order to model the effect of tumour location. Shape features, first-order histogram statistics and texture features (‘intensity radiomics’) were calculated for each lesion and averaged over the whole disease. Intra-lesion heterogeneity was assessed by contracting the lesion contours and calculating the ratio of radiomics features before and after the contraction (‘rim radiomics’). Similarly, the external context of the lesions was assessed by calculating the ratio of radiomics features before and after dilating the contours (‘peripheral radiomics’). Finally, we also defined a series of binary variables to describe additional radiological findings (‘semantic features’): ascites and pleural effusion were assessed manually, and we used a previously developed automated tissue-specific sub-segmentation tool to identify hypodense (cystic/necrotic spaces) and hyperdense (calcifications) lesion parts within the manual segmentations^25^.

**Fig. 2:**
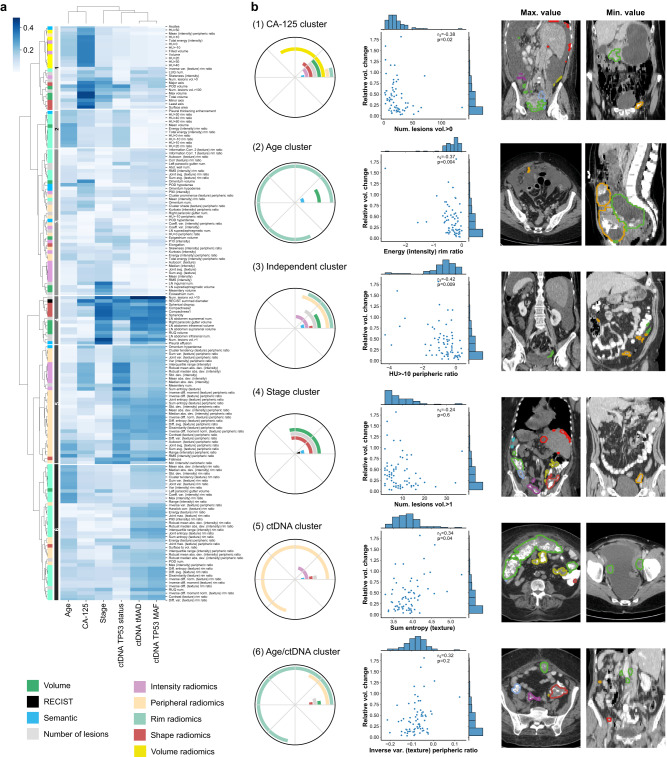
Analysis of the correlations between radiomics and non-imaging features. **a** Spearman correlation coefficients between imaging (rows) and clinical and biological features (columns), both clustered using a hierarchical approach, using the training cohort. **b** Composition and characteristics of the six identified imaging feature clusters. Polar plots indicate the relative contribution of the different classes of imaging features. Scatter plots show the feature of each cluster with the highest Spearman correlation with volumetric treatment response. Each features is illustrated by displaying one slice from the patient with the maximum value (left), and one from the patient with the minimum value (right). Source data are provided as a Source Data file. *p* values are two-sided and corrected for multiple comparisons.

We found that imaging features grouped into six distinct clusters (Fig. 2a). Cluster 1 was associated with baseline CA-125 levels (*r*_median_ = 0.34) and contained mostly lesion volume metrics. This is consistent with previous work suggesting that CA-125 correlates with lesion volume^26^. Clusters associated with ctDNA features were generally dominated by features quantifying lesion heterogeneity and context. Cluster 5 was primarily associated with ctDNA *TP53* status (*r*_median_ = 0.25), and contained predominantly peripheral radiomics features, which quantify lesion context. Cluster 6 was associated with ctDNA *TP53* MAF and tMAD (*r*_median_ = 0.20 and 0.19, respectively), and contained predominantly rim ratio radiomics features, which provide information on intra-lesion heterogeneity.

Cluster 4 was highly correlated to stage (*r*_median_ = 0.36), and was composed of a mixture of features related to shape, volume, and number of lesions, which quantify the disease burden. This is consistent with the definition of FIGO stage, which relies on the assessment of the extent and spread of the disease^27^. Cluster 2 was associated mostly with age (*r*_median_ = 0.23) and contained almost exclusively rim ratio radiomics features, which provide information about intra-tumour heterogeneity. The remaining group (cluster 3) was formed by a heterogeneous mixture of features, and did not associate with any biological or clinical feature.

These results indicate that some of the information from global biomarkers such as stage, CA-125 or ctDNA can also be captured in multi-lesion radiomic features that quantify the extent, spread, heterogeneity and context of the disease. In addition, as shown in Fig. 2b, clusters 1, 2, 3 and 5 contain imaging features that are significantly correlated with volumetric response to treatment after multiple comparison correction. Cluster 3 has negligible associations with either biological or clinical features, demonstrating that radiomics features can also contribute unique information to integrated radiogenomic predictive models.

### Integrative ensemble model predicts volume response to neoadjuvant chemotherapy

We built an integrative radiogenomic machine learning framework to predict response to neoadjuvant chemotherapy, called *IRON* (Integrated Radiogenomics for Ovarian Neoadjuvant therapy). The framework is a robust ensemble of three machine learning pipelines, each of which includes a classifier (elastic net, support vector regression, or random forest) preceded by collinearity reduction and feature selection steps (see Methods). To further prevent overfitting, models were trained using a 5-fold cross-validation setup that was repeated five times with different seeds. To capture the behaviour of the whole metastatic disease, we defined the response metric as the relative change in total disease volume, which was also correlated with surgical debulking status (Supplementary Fig. S5).

**Fig. 3:**
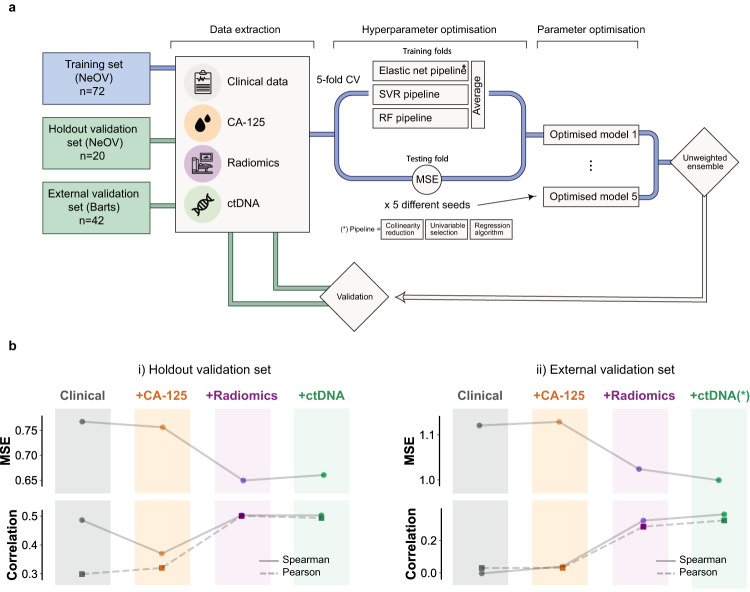
Training scheme and validation results for the *IRON* machine learning framework. **a** Schematic of the machine learning framework for model training and validation. **b** Validation of the discriminative power of predictive models in the hold-out (left) and external validation cohorts (right), for models containing, from left to right, clinical, clinical+CA-125, clinical+CA-125+radiomics, and clinical+CA-125+radiomics+ctDNA features, respectively. The metrics are mean square error (MSE, top) and Spearman (continuous) or Pearson (dashed) correlation (bottom). The magnitude of the mean squared error (MSE) is comparable to the standard deviation of the volumetric response (Supplementary Table S9). ctDNA values in the external validation cohort were imputed using training set averages. Source data are provided as a Source Data file.

We used three distinct datasets: a training set (NeOV *n* = 72), a hold-out validation set (NeOV *n* = 20) and an independent validation set (Barts *n* = 42). We trained four models by successively adding clinical and molecular features: (i) age, FIGO stage, and treatment; (ii) CA-125; (iii) radiomics features; and (iv) ctDNA, (Fig. 3a and Supplementary Data 1). All features used in the model were extracted from data obtained at diagnosis. Once trained, the four models were frozen and tested on the hold-out and external validation sets.

**Fig. 4:**
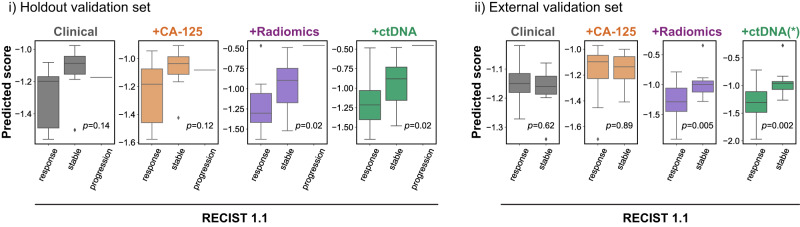
Using the *IRON* framework to predict responding, stable and progressive disease according to RECIST 1.1. Validation of the ability of predictive models to describe RECIST 1.1 response in the hold-out (left, *n* = 20) and external (right, *n* = 42) validation cohorts, for models containing, respectively, clinical, clinical+CA-125, clinical+CA-125+radiomics, and clinical+CA-125+radiomics+ctDNA features. Boxes indicate the upper and lower quartiles, with a line at the median. Outliers are shown as circles and identified via the interquantile range rule. *p* values are obtained using the point biserial coefficient and are two-tailed. ctDNA values in the external validation cohort were imputed using training set averages. Source data are provided as a Source Data file.

On the hold-out validation set, adding successive features resulted in a gradual MSE reduction of 15% for the model without ctDNA, and 14% after integrating ctDNA (Fig. 3b and Supplementary Table S8). Only the two models that included radiomic features were able to produce response scores that were significantly correlated with the observed volume response (Spearman *r* = 0.5, *p* = 0.02, Fig. 3b and Supplementary Table S8). Although the models were not trained to predict RECIST 1.1, we observed that the scores predicted by the integrated radiomics model were able to correctly rank the three RECIST 1.1 response groups (*p* = 0.02, Fig. 4). Similarly, we found that the model was able to accurately predict which patients were most likely to undergo delayed primary surgery, but it was not able to predict surgical debulking status (Supplementary Fig. S5).

We tested the generalisability of the models in an independent external cohort (Barts cohort, *n* = 42). As this cohort did not have ctDNA testing performed, we imputed ctDNA measurements to the training set averages. The clinical and CA-125 models did not achieve significant performances in terms of Spearman correlation with response. The radiomics and ctDNA models both achieved significant performance at a similar level (*r* = 0.32, *p* = 0.04 and *r* = 0.32, *p* = 0.02 respectively, Fig. 3b and Table S8). For volume shrinkage thresholds corresponding to clinically relevant criteria (RECIST, WHO, spherical-volume and ellipsoidal-volume response), the full model achieved an average area under the receiver-operating characteristic curve (AUC) of 0.7, compared to an average of 0.5 for the clinical and CA-125 models (Supplementary Fig. S7). When assessed against the RECIST classification labels, the full model achieved an AUC of 0.8, similar to the radiomics model (0.78), compared to 0.47 and 0.50, respectively for the clinical and CA-125 models.

**Fig. 5:**
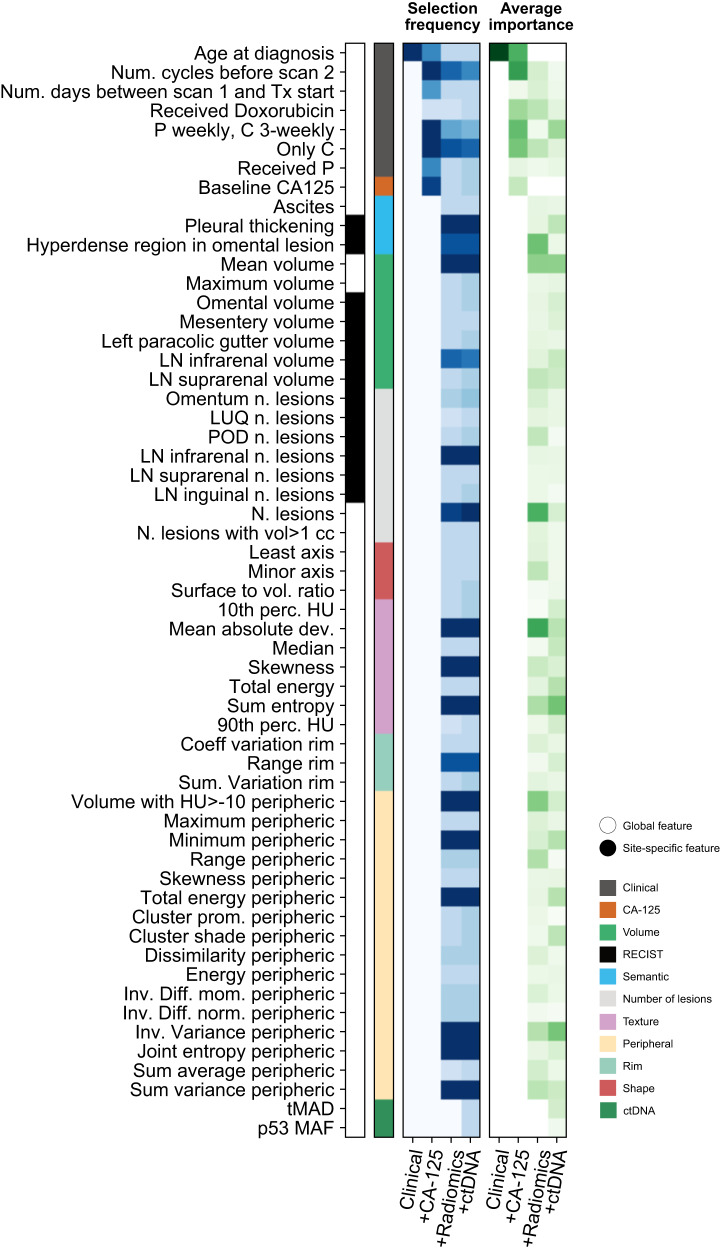
Feature importances in the fully integrated *IRON* radiogenomic model. Importances of the features used by the predictive models. The first (blue) heatmap illustrates the selection frequency. The heatmap shows the number of times that a given feature was selected in a model. The different columns correspond to different models with increasing, cumulative numbers of input features. As the optimisation is repeated five times, the range of the selection frequency is 0–15 (three algorithms in the ensemble times five repetitions). The first (green) heatmap illustrates the averaged, normalised feature importances for the elastic net and random forest components of the models. Importances are defined from the feature coefficients for the elastic net regression, and from impurity-based Gini importances for random forest. Source data are provided as a Source Data file.

We studied the relative contribution of the features used by the final models in two different ways. Firstly, we evaluated the selection frequency for each feature (Fig. 5a). We found that the treatment regimen and the number of sessions of chemotherapy before the second scan were consistently selected across models. We also found that CA-125 was used in models that did not include radiomics, but was dropped from radiomics integration models. Semantic features, in particular pleural thickening and the presence of a hyperdense region in an omental lesion, were selected across all the relevant models. The only volumetric features selected consistently were mean volume and the volume of the infrarenal lymph nodes. A small and consistent number of radiomics features was also selected, most of them belonging to the category of features quantifying lesion context. We found that volume-related radiomic features play an important but not essential role, as the model is still predictive if they are removed during inference. Secondly, we quantified relative feature importances within the models (Fig. 5). We found that most of the models integrating a large number of features tend to be more dense, with features sharing similar, lower importance levels. Features that tended to have larger importance were generally consistent with those that had the highest selection frequency, as can be observed by comparing the two panels in Fig. 5.

## Discussion

The clinical presentation of HGSOC is with complex, highly heterogeneous disease that is invariably metastatic throughout the abdomen. The accompanying genomic and cellular heterogeneity has impeded therapeutic progress and strongly suggests that understanding response to treatment must involve the integration of data from different sources and scales. We have shown that an integrated radiogenomic machine learning model based on baseline multi-scale data predicts volumetric response to NACT (*p* = 0.04, external validation cohort). Our results show that radiomics features are critical for the prediction, and that models that do not include radiomics fail to predict response. We also revealed site-specific patterns of differential response and correlation of radiomics features with ctDNA detection.

The field of radiomics has grown exponentially in recent years, showing great promise across tumour sites and endpoints^28^. At the same time, radiomics has been criticised for lack of robustness and reproducibility, as well as lack of biological interpretability^29,30^. Our study shows that both problems can be overcome by the right design choices.

We made robustness a design priority for our predictive framework, *IRON*, which included strong feature selection, model ensembles on multiple levels, and repeated re-shufflings of the data to avoid biases. In addition, we trained the model on a dataset with heterogeneous imaging parameters^31^; and we included new families of imaging features based on ratios between different volumes of interest, which were designed to partially cancel out such biases. We also curated an independent cohort from a different institution for validation, which we were able to perform successfully.

Our feature definitions were also designed to improve interpretability: the ratio features are not only robust to imaging parameters, but they also helped us to explore internal heterogeneity (rim features) and the external context of the lesions (peripheral features). Interestingly, we found that ratio features were the most frequently selected in the final models. This result is in line with previous work that found that qualitative radiological features describing the edges of peritoneal disease (nodular, diffuse, or mixed) were significantly associated with CLOVAR subtype^32^ and BRCA mutation status^33^. The concept of peritumoural radiomics has been explored before in breast, lung, and liver cancer^34–37^. The periphery of tumours and the adjacent peritumoural stroma are characterised by molecular and cellular changes directly related to tumour biology. Neoangiogenesis and fibroblast invasion have been demonstrated in the tumour periphery of ovarian cancer^38^, whereas characteristic immune cell populations have been identified in the peritumoural stroma^39,40^. Our findings motivate the development of more detailed studies focusing on the boundary regions of ovarian cancer lesions, demonstrating that data-driven radiomics analyses can support biological hypothesis generation.

Our study confirms previous observations that the presence of ctDNA is correlated with volume of disease at the start of treatment in HGSOC patients^20^. The integration of clinical data into our models also yielded important insights, as features describing the type of NACT and its timing were consistently selected by our models. The ICON8 study (to which part of the NeOv cohort was recruited) showed that PFS in patients with ovarian cancer undergoing NACT was unaffected by the administration regimen of paclitaxel (3-weekly as is standard or in a dose-dense weekly regimen)^3^. However, the feature describing the mode of administration (weekly versus 3-weekly) was consistently selected by our prediction models. There are several possible explanations for this, including the limited size of our dataset, or a possible non-linear interplay with some of the other variables in the model. Alternatively, the administration mode could potentially be a factor that affects response to NACT but not survival. This is still clinically important, since disease extent after NACT affects resectability of the disease at DPS and, therefore, perioperative morbidity and operating time^41^.

Previous studies have explored the value of radiogenomic data integration for HGSOC^14,42^. Their analyses focus on immediate primary surgery and localised biomarkers (pathology and genomics from a single biopsy; radiomics from single lesions), whereas we focus on neoadjuvant therapy and analyse global biomarkers capturing metastatic disease (circulating tumour DNA and multi-site radiomics). We enriched our data set by segmenting the full disease burden and including radiomics of all abdominopelvic tumour locations, reaching a median of 18 volumes of interest per scan in the training set. Our analysis showed that response to NACT in HGSOC varies spatially: lesions in the ovaries and/or pelvis had poorer response than those in the omentum. Our analysis also integrates imaging features for all of the abdominopelvic disease with blood-based biomarkers, including ctDNA and CA-125. We showed that global tumour burden and volume of pelvic and ovarian tumours at baseline are significantly correlated with both CA-125 and ctDNA *TP53* MAF. However, our analysis of volumetric data also showed that ctDNA signal was most strongly correlated with the volume of disease in the ovaries and/or pelvis, suggesting that the simultaneous reading of ctDNA and CA-125 at baseline could play a role in helping determine disease spread and response in the diagnostic setting. This variation could be due to potential differences in ctDNA release kinetics or to genomic heterogeneity between disease sites.

Our study has several limitations. Firstly, the response metric (relative total volume change) treats the disease as a single entity, ignoring the subtle interplay between the pelvis/ovaries and the omentum. Cell-of-origin analysis via mutation, methylation or fragmentomics to infer contribution from specific cell types to the circulating free DNA pool could help to predict specific volume reductions. Secondly, the importance of some data streams is driven by the specific features that were included in the model. Indeed, given the correlation between ctDNA and total disease volume, and the higher dimensionality of the collections of clinical and radiomics features, it is not surprising that we did not find any additional value in adding ctDNA based biomarkers to our integrated models. Our study was limited to ctDNA t-MAD, *TP53* MAF and *TP53* mutation status. A more sophisticated analysis of ctDNA, applied on larger multi-institutional datasets, and measured longitudinally across different time points, may find that there are other complementarities between ctDNA and imaging data^43–46^. Another important limitation of our results is the size of the datasets used. This may have impacted the added value of ctDNA features and reduced the strength of the radiomic correlations, which are often borderline significant. Our conclusions need to be validated in larger cohorts as the next step in their development.

Our dataset also lacked detailed quantification of known biomarkers such as *BRCA1/2* or *CCNE1* mutations, homologous recombination deficiency, or molecular subtypes as evaluated from gene expression or copy number data, which are likely to be important factors and should be incorporated into future models^47–55^. For a small subset of samples in the hold-out validation cohort that did have *BRCA1/2* status information, we did not find that it had an effect on either the response or the errors of the prediction (Supplementary Fig. S6). Detailed investigations regarding histopathological features such as the presence of necrosis, extent of tumoural fibrosis, or quantification of reactive stroma, were also beyond the scope of this study. Adding extensive molecular profiling and computational pathology in the future will not only potentially improve the performance of the model, but also enable detailed biological interpretation of the main drivers behind response.

One potential barrier for clinical implementation of this work is that manual segmentation for volumetry is time consuming. However, accurate and highly adaptable deep learning models for automatic segmentation are being rapidly developed across cancer types, facilitating the integration of volumetry into clinical workflows as well as large-scale radiomics computation^56,57^.

In conclusion, our study is a proof-of-principle for the integration of radiogenomic data to describe and predict the response of HGSOC patients to NACT. We demonstrate that the systematic multi-scale integration of standard-of-care biomarkers provides critical predictive power and important insights into the complex spatial configuration of the disease. Further clinical development of *IRON* could have significant impacts as a stratification tool in clinical and experimental settings—for example avoiding delays in surgery for patients who are unlikely to respond to chemotherapy—and could bring forward a new generation of clinical trials for HGSOC, with rapid, effective endpoints that improve and expedite the discovery of new therapies.

## Methods

### Patient cohorts

This study was performed in accordance with the principles of the 1964 Declaration of Helsinki and its later amendments or comparable ethical standards. Written informed consent was obtained from all patients prior to any study related procedures.

Two patient cohorts were used in the study. The main cohort (the ‘NeOV’ cohort) was randomly split into a training set and a hold-out set. The training set was used to train the machine learning models, and as a discovery dataset for univariable analyses. The hold-out set was set aside and used only to validate the model predictions. A second dataset (the ‘Barts’ cohort) was used for external validation.

For both data sets, patients had a confirmed histopathological diagnosis of HGSOC and were treated with neoadjuvant chemotherapy before delayed primary surgery. All patients within the main data set were treated at Cambridge University Hospitals NHS Foundation Trust between 2009 and 2020 and were recruited into a prospective clinical study approved by the local research ethics committee (REC reference numbers: 08/H0306/61). All patients within the Barts data set were treated at Barts Health NHS Trust between 2009 and 2018 and recruited into prospective clinical study approved by the local research ethics committee (IRAS reference numbers: 243824).

Patients were identified and included in the analysis based on the availability of at least two CT scans at baseline and after NACT. Additionally, for the NeOV cohort at least one baseline plasma sample for ctDNA assessments was required.

### Clinical data

Data regarding patient demographics, treatment, and disease were collected from the patient electronic medical records including notes from multidisciplinary team discussions (MDTs). PFS was defined as the time between histopathological diagnosis and first radiological evidence of progression or recurrence. Where progression date was unclear from radiology reports alone, (e.g. successive imaging studies with subtle/mixed changes), clinical interpretation of progression was incorporated in PFS date calling (e.g. documentation of breaking bad news to patients, treatment decisions for subsequent line therapy). OS was defined as the time from diagnosis to death. Stage was determined using the International Federation of Gynaecology and Obstetrics (FIGO) criteria for ovarian cancer^27^.

#### Management

The management of all patients in the study including indications for surgery were discussed and decided upon within MDTs as per the UK National Health Service (NHS) guidelines. Surgeries were performed through a midline laparotomy by a team specialised in surgical gynae-oncology aiming to achieve total macroscopic tumour clearance. Overall, *n* = 107 patients were treated with platinum-based chemotherapy in combination with paclitaxel, while 23 received carboplatin as a monotherapy. Patients were considered to have received weekly chemotherapy if the average time interval between doses ranged between 6 and 10 days, 3-weekly if the average time interval was between 18 and 24 days, and irregular otherwise. Of those receiving combination therapy, 81 patients received carboplatin and paclitaxel 3-weekly, 18 patients received carboplatin three-weekly and paclitaxel weekly, 2 patients received carboplatin and paclitaxel weekly, and the rest received the treatment at irregular intervals. In addition, 4 patients were treated with Doxorubicin. Supplementary Table S3 shows the breakdown for the three datasets.

#### BRCA status

Germline *BRCA1* and*BRCA2* mutational status was determined for 45 patients in the training cohort and 15 patients in the internal hold-out cohort. The remaining cases reflect historical practice of not testing patients above the age of 70.

### ctDNA

Blood samples were collected before initiation of treatment with chemotherapeutic agents. DNA was extracted from plasma (1.2–4 ml) using QIAvac 24 Plus vacuum manifold and the QIAamp Circulating Nucleic Acid kit (Qiagen), or with QIAsymphony (Qiagen) as per manufacturer instructions. DNA quantification was performed using Qubit dsDNA broad-range or high-sensitivity assay kits and the Qubit Fluorometer (Thermo Fisher Scientific). Tagged-amplicon deep sequencing DNA libraries were prepared as described by ref. ^58^. Following purification with AMPure XP magnetic beads (Beckman Coulter Life Sciences), 10nM libraries were quantified using Agilent Bioanalyzer and Agilent DNA 1000 kit or Agilent TapeStation and ScreenTape D1000 (Agilent Technologies) according to manufacturer instructions, and pooled for sequencing on MiSeq, HiSeq 2500 or HiSeq 4000 (Illumina).

Shallow whole genome DNA libraries (10 million reads per sample) were prepared using the ThruPLEX DNA-Seq kit (Takara) and purified with AMPure XP magnetic beads (Beckman Coulter Life Sciences). 10nM libraries were quantified using Agilent D5000 ScreenTape System or Roche KAPA library quantification kits and pooled for sequencing on HiSeq 4000 (Illumina) in paired-end 150-base pair mode. On removing adaptor sequences, shallow whole genome sequence reads were aligned to the 1000 Genomes Project version of the unmasked human reference genome GRCh37 using the BWA-MEM alignment software^59^. Somatic copy number analysis was performed using CNAclinic^60^ to generate trimmed Median Absolute Deviation from copy-number neutrality (t-MAD) scores as previously described^21^.

Demultiplexed TAm-Seq reads were aligned to the GRCh37 reference genome by amplicon, and mutations called where non-reference alleles met probability criteria in both replicates, as previously described^58^. Samples lacking mutation calls were manually curated using the Integrative Genomics Viewer.

### Imaging protocol

Although adnexal masses are most frequently detected on ultrasound (US), CT is a universal tool for the detailed staging of patients with adnexal masses and is crucial for treatment planning as it maps the disease for surgical debulking and helps stratify patients for primary debuling surgery or neoadjuvant chemotherapy. US suffers from high observer-dependency and does not provide images covering the entire pelvis and abdomen as CT does. Therefore, CT was chosen as the imaging modality for this study. Clinically requested contrast-enhanced venous phase CT scans covering the abdomen and pelvis (with or without the chest depending on the clinical request and imaging findings) were either acquired at Cambridge University Hospitals NHS Foundation Trust (CUHNHSFT) or in other institutions across the UK and then imported into the picture archiving and communication system (PACS) at CUHNHSFT. Therefore, different manufacturers and scanning protocols were used. Baseline scans were acquired between 0 and 14 weeks before initiation of neoadjuvant chemotherapy and post-treatment scans were acquired for response assessment after 1.6–5.8 months of treatment. All scans were initially identified on the local PACS and then fully anonymised for further study-related processing.

### Data processing

The code and data used in this study are available at https://github.com/micrisor/OvarianIntegration^61^.

#### Image segmentation and labelling

On axial images reconstructed with a slice thickness of typically 5 mm (Supplementary Table S4), and pixel spacings ranging between 0.053 and 0.095, using abdominal soft tissue window settings, all cancer lesions were segmented semi-automatically by a board-certified radiologist with ten years of experience in clinical imaging, using Microsoft Radiomics (project InnerEye; Microsoft, Redmond, WA, USA). The volumes of interest (VOIs) were annotated for their anatomic location: omentum, right upper quadrant, left upper quadrant, epigastrium, mesentery, right paracolic gutter, left paracolic gutter, ovaries & pelvis, infrarenal abdominal lymph nodes, suprarenal abdominal lymph nodes, inguinal lymph nodes, supradiaphragmatic lymph nodes, other chest lymph nodes, parenchymal liver metastases, and lung metastases. Cystic and solid tumour parts were included in these segmentations. Automated sub-segmentation of hyperdense/calcified, hypodense/cystic or fatty and intermediately dense/solid tissue was performed for omental lesions and lesions of the ovaries and pelvis using a previously described and validated technique^25^. Baseline and follow-up CT scans were evaluated according to RECIST 1.1 for response assessment^62^. Pleural effusions and ascites were assessed semiquantitatively (0 = none, 1 = trace, 2 = less than 5 cm when measured perpendicularly to chest/abdominal wall, 3 = 5 cm or more when measured perpendicularly to chest/abdominal wall).

#### Radiomics features

VOIs drawn manually were split into connected components using MATLAB’s *bwlabeln* function with a three-dimensional connectivity of 26, which assumes that voxels are connected if their faces, edges, or corners touch. Voxels with intensities below -100 HU were removed from the radiomics calculations. Radiomics features were extracted using the IBSI-calibrated CERR Radiomics toolbox^63^ (December 2018 version, GitHub hash: 5974376be7103d5c3831690c62aa721fc784d949), including shape, intensity-volume histogram, first-order, and Haralick texture features (see Supplementary Data 1 for the full list). Intensity-volume histogram features, inspired by the Vx features commonly extracted in radiotherapy dose-volume histograms, corresponded to the volumes spanned by voxels above a certain intensity value (denoted ‘HU > x’ in Supplementary Data 1). To calculate Haralick texture features for each lesion, co-occurrence matrices for 100 grey levels (up to a maximum of 1000 HU) were computed independently for each direction along 2D slices and averaged. To calculate the rim and peripheral radiomics features, for each VOI two copies were created by eroding and dilating the contours by 0.4 cm along the 2D slices. The value of the margin was chosen in order to capture slices of at least 1 cm diameter. Erosion and dilation were achieved by convolving the contour with a circular mask of the desired margin. Ratio features were computed by dividing the results obtained from the eroded and standard volumes (rim features), or the dilated and standard volumes (peripheral features). Shape features were not included in the ratios. Once standard and ratio features were calculated for each lesion, a single value was extracted for the whole patient by taking the unweighted mean of all lesions.

#### Other imaging features

In contrast to texture features, which we averaged across lesions, we did use the volume and the number of lesions in each of the anatomic locations as individual features. We also computed the mean, maximum, and total volume, as well as the number of lesions with volume bigger than 0, 1, 10 and 100 cm^3^. In addition, we defined four binary features that indicated whether or not there were hypodense or hyperdense regions in either omental or pelvic/ovarian lesions. Ascites and pleural effusion were used as defined by the radiologist, as explained above (section 14, Radiological image analysis).

#### Clinical features

Chemotherapy regimens were extracted from the clinical records. We recorded whether the patient had received any Carboplatin, Paclitaxel or Doxorubicin in three binary variables. Mean periods were calculated by averaging the time intervals between sessions. We defined weekly regimen as having a mean period of 6–10 days, both included; and 3-weekly as having a mean frequency of 18–24 days, both included. Typical combinations included weekly Paclitaxel and 3-weekly Carboplatin; both weekly; and both three-weekly (Supplementary Fig. S1). These combinations were, therefore, encoded in binary variables. We also recorded whether patients had received Carboplatin only in a binary variable. FIGO stage was encoded by assigning ordinal numbers to in order of aggressiveness, from 1 for stage 1A to 10 for stage 4B. The exact mapping is listed in Supplementary Table S5. CA-125 values were also extracted from the clinical records, with the measurement closest to the beginning of treatment being used for analysis. Performance status was only available for a subset of the patients in the training set, and it did not correlate with volumetric response (Supplementary Fig. S2). It was, therefore, not included in the predictive models.

### Statistical analysis

#### Tumour burden correlations

Correlations were calculated using Spearman correlation coefficient and *p* values were corrected using the Benjamini–Hochberg procedure as implemented in the scikit-learn Python package^64^. The total tumour burden affecting lymph nodes was calculating by combining the lesions found in infrarenal, suprarenal, inguinal, supradiaphragmatic, and chest lymph nodes. The difference between the relative volume change in the omentum and in the ovaries/pelvis was quantified using the Mann-Whitney *U* test (two-sided). The difference in relative volume change for patients with different BRCA status was also quantified using the Mann-Whitney *U* test (two-sided).

#### Imaging clusters

To identify clinically or biologically meaningful clusters of radiomics features, we clustered their Spearman correlation coefficients used a hierarchical clustering approach. The optimal number of imaging clusters was obtained by maximising their correlation with any of the clinical and biological features. To do this, we calculated the maximum Spearman correlation coefficient between each cluster and any of the biological/clinical features, and averaged the result across all clusters (Supplementary Fig. S4a). The metric reached a plateau at six clusters, which was therefore chosen as the optimal number (Supplementary Fig. S4b). Clusters were cross-checked using non-negative matrix factorisation. We matched the number of clusters to 6, used a coordinate descent solver and an initialisation based on non-negative random matrices, following the scikit-learn implementation. The most predictive feature in each cluster (Fig. 2b) was chosen in terms of its Spearman correlation with volumetric treatment response. *p* values were corrected using the Benjamini-Hochberg procedure.

### Machine learning models

#### Endpoint

Models were trained to predict the relative volumetric response,

1 $${{{{{{{\rm{Response}}}}}}}}=\log \left(\frac{\mathop{\sum }\nolimits_{i}^{{N}^{{\prime} }}{v}_{i}^{1}}{\mathop{\sum }\nolimits_{i}^{N}{v}_{i}^{0}}\right),$$

where ${v}_{k}^{0}$ is the volume of the *k*th lesion found at the pre-treatment time point, ${v}_{k}^{1}$ is the volume of the *k*th lesion found at the post-treatment time point, *N* is the number of lesions at the pre-treatment time point, and ${N}^{{\prime} }$ is the number of lesions at the post-treatment time point.

#### Training

We created a machine learning framework to predict response to chemotherapy, evaluated on the basis of relative total volume change. All features used in the model were extracted from data obtained at diagnosis. We used the NeOv training set to train and optimise the models. Once trained and frozen, we evaluated the models in the internal hold-out set and in the external validation set. We used an increasing number of features, in order of general availability. We started with clinical features (age, stage, and 9 treatment features); then added baseline CA-125; then imaging features (164 features); and finally ctDNA (3 features). For each combination we retrained the framework and derived a new model. The full list of features can be found in Supplementary Data 1.

The predictions were based on an unweighted ensemble regressor^65^. The ensemble included three different machine learning algorithms: an elastic net, a support vector regressor with a radial basis function (RBF) kernel, and a random forest, all of them coded in Python using the scikit-learn package^64^. Each algorithm was embedded in a scikit-learn pipeline with three pre-processing steps, namely collinearity reduction, z-score standardisation, and univariable feature selection. Collinearity reduction removed all features with a mutual Pearson correlation above 0.95, retaining only the one with the highest correlation with the response variable. The feature selection step removed all features that were not ranked within the top *k* according to their *F* value. The scores produced by the three pipelines were averaged to form the prediction.

We used a five-fold cross validation setup to optimise model hyperparameters in the training set, covering the hyperparameter ranges shown in Supplementary Table S6. The optimisation was based on a randomised search in the hyperparameter space to optimise mean square error (MSE). Once the optimal hyperparameters were found (Supplementary Table S7), we determined model parameters by re-fitting the model to the entire training set. To increase model robustness, we repeated this process five times with five different cross-validation seeds. The five resulting optimal models were combined to form the final ensemble, in which the prediction is simply the average of the five predicted scores. In this regard, our ensemble setup has two different tiers: the randomisation tier (five seeds), and the algorithmic tier (three regressors acting in parallel for each seed). The modelling framework is completely agnostic to cancer type and is generalisable to other applications.

#### Validation

We validated the models on the hold-out internal validation set (*n* = 20) and the external validation set (*n* = 42). The external validation set did not have ctDNA data available, so the corresponding features were set to a constant value corresponding to the training set averages as a trivial imputation mechanism. To quantify the calibration of the models, we computed the MSE. To quantify the discriminative power of the models, we computed the Spearman correlation coefficient and *p* value between the predicted and observed response scores. To estimate a potential classification power on the external validation cohort, we evaluated the volumetric thresholds corresponding to RECIST 1.1 criteria (30% reduction), WHO criteria (50% reduction), spherical-volume (65% reduction), and ellipsoidal volume (30% reduction) and computed the corresponding area under the receiver-operating characteristic curve (AUC). Finally, we evaluated whether the predicted scores were also able to rank patients into the different RECIST 1.1 categories using the *p* value associated with the point biserial correlation coefficient and the AUC for responder vs. stable disease.

#### Feature importance

We evaluated feature importance in two different steps. First, we computed the frequency with which features were selected after the collinearity reduction and univariable selection steps. We repeated the process for each of the three algorithms and each of the five cross-validation seeds, which means that features could be selected between 0 and a maximum of 15 times, as seen in Fig. 5. The table in Fig. 5 displays only features that were chosen at least three out of five times in each cross-validation loops, for robustness. Second, we computed the importance of each individual feature within the regression algorithm. This was only possible for the elastic net, where we used the feature’s coefficients, and the random forests, where we used impurity-based feature importances. The results were averaged across the five seeds and the two algorithms, with the table in Fig. 5 displaying only features that were chosen at least three out of five times in the cross-validation loops, as before. Finally, we assessed the effects of volume-related features, including the two most frequently selected volume features (mean volume and volume of infrarenal lymph nodes), and the two clusters with important contributions from volume features (cluster 1 and cluster 4). Features were removed at inference stage, and replaced with the corresponding average calculated from the training set. Removing features that drive the prediction would result in a significant change in performance, which can be either positive or negative depending on whether the corresponding features in the training and testing sets are mutually calibrated.

### Supplementary information

Supplementary Information

Description of Additional Supplementary Files

Supplementary Dataset 1

### Source data

Source Data

## Data Availability

The clinical, genomic, and radiomics data generated in this study and used to train predictive models have been deposited in a Github repository at https://github.com/micrisor/OvarianIntegration^61^ with 10.5281/zenodo.8152137. The remaining data are available within the Supplementary Information and Source Data files. Source data are provided with this paper.

## Code availability

The code and data used in this study are available at https://github.com/micrisor/OvarianIntegration^61^ with 10.5281/zenodo.8152137.
