# Supplementary material for: Integrated radiogenomics models predict response to neoadjuvant chemotherapy in high grade serous ovarian cancer
Source: Nat Commun. 2023 Oct 24;14:6756. doi: 10.1038/s41467-023-41820-7 (PMC10598212; doi:10.1038/s41467-023-41820-7)
Supplement: Supplementary file 1 — Supplementary Information [file 41467_2023_41820_MOESM1_ESM.pdf]

A. Supplementary figures

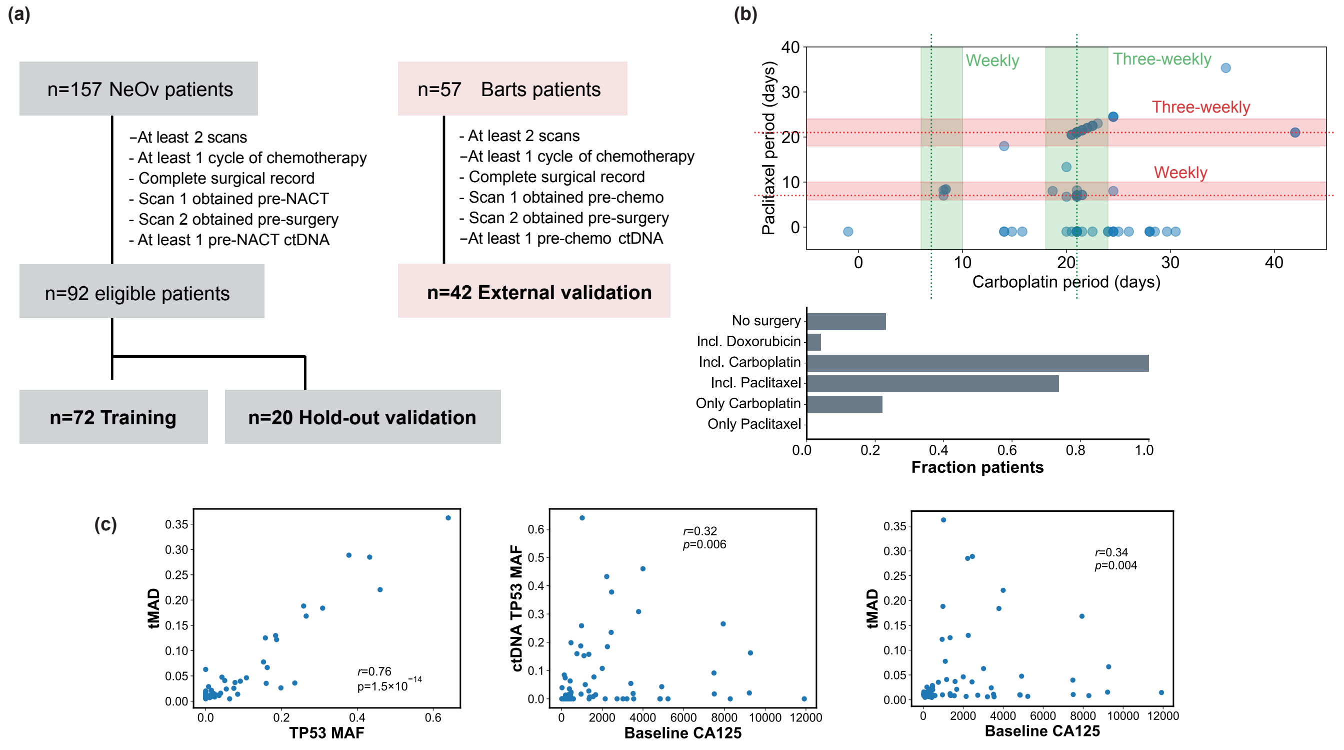

Supplementary Fig. 1. **a** Flowchart of the datasets used in the analysis. **b** Paclitaxel and carboplatin administration frequencies for patients in the NeOv cohort. Bands indicate the range of patients who are considered to have received either weekly, three-weekly, or other treatment frequency. **c** Scatter plots illustrating the Spearman correlations between the blood-based biomarkers (two-sided *p* values , Spearman correlation coefficient). Only the training dataset was used for this analysis.

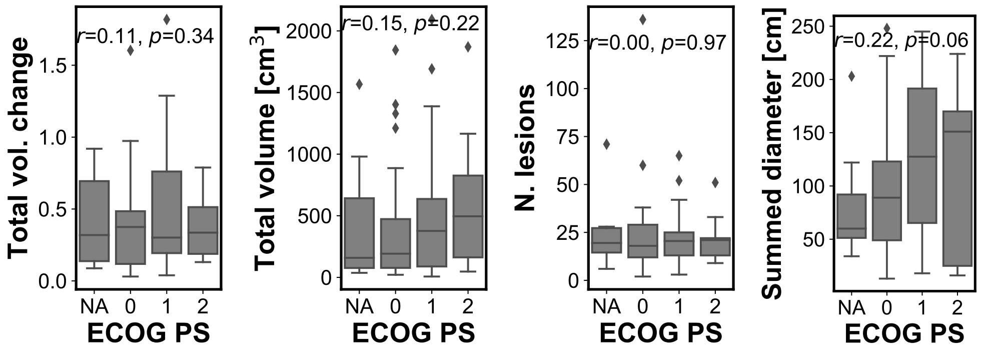

Supplementary Fig. 2. Relationship between performance status a nd, from left to right, total volume change, total volume at baseline, number of lesions at baseline, and summed diameters of the RECIST lesions at baseline, evaluated in the training set (n=72). There is an ascending trend in the total volume and summed diameter, but none of the Spearman correlations are significant (two-sided *p* values , Spearman correlation coefficient). Boxes indicate the upper and lower quartiles, with a line at the median. Outliers are shown as circles and identified via the interquantile range rule.

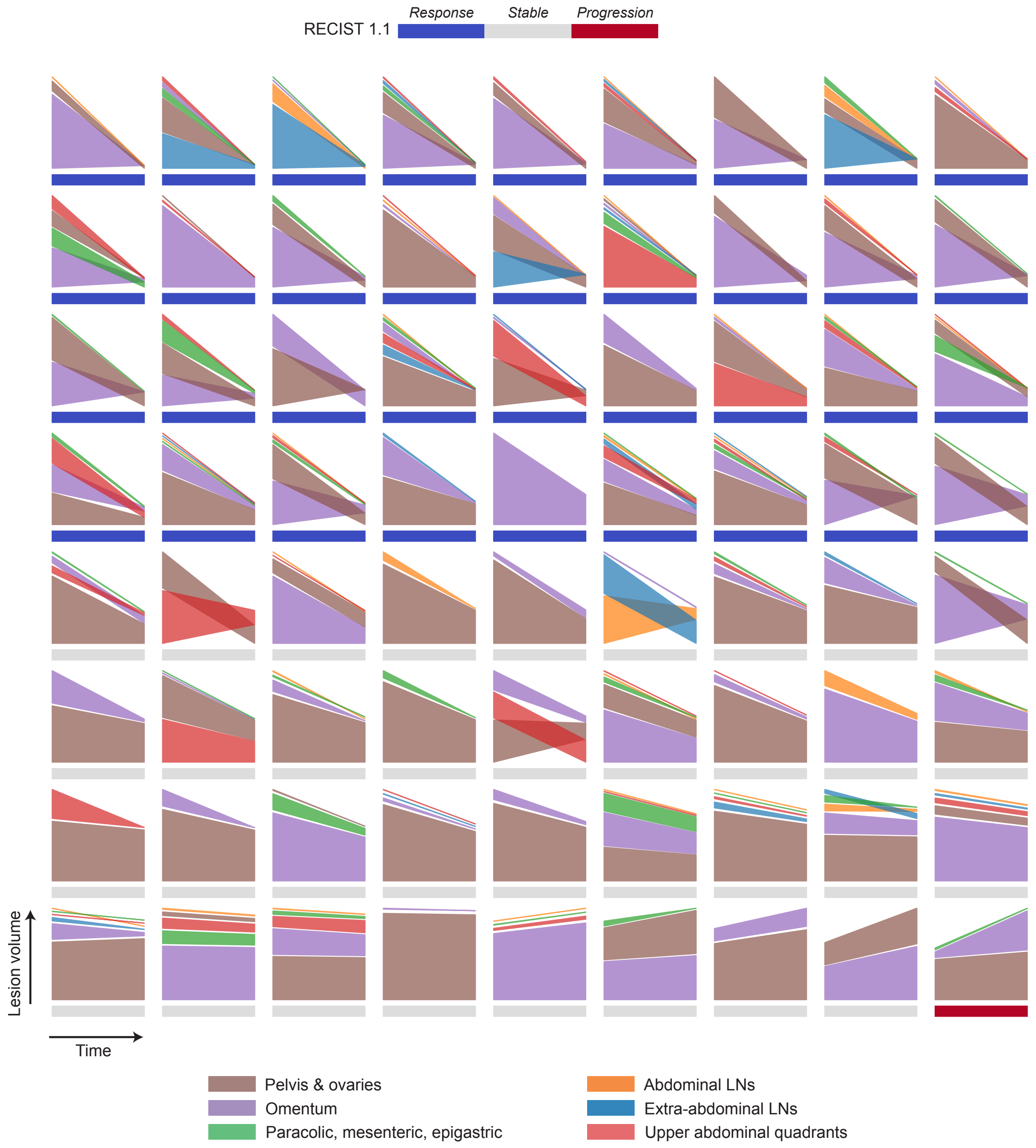

Supplementary Fig. 3. Site-specific change in volume for each of the patients in the training dataset. Patients are ordered as a function of the total change in volume.

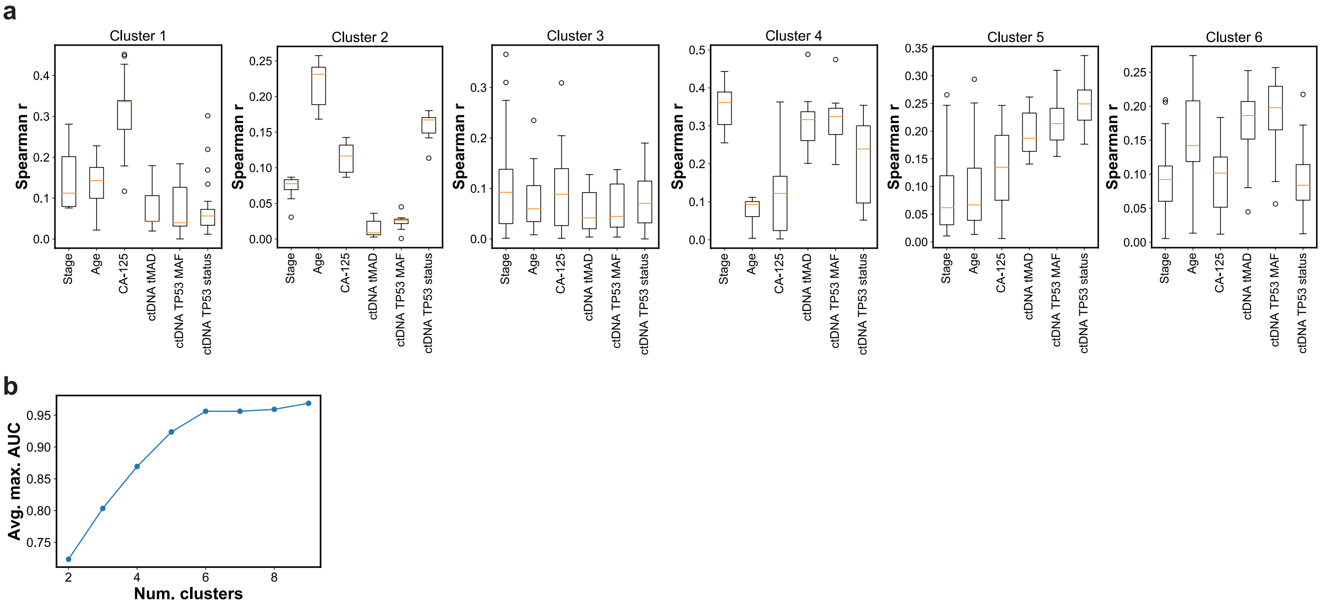

Supplementary Fig. 4. a Correlation between each of the imaging clusters and clinical features and blood biomarkers in the training cohort (n=72). Boxes indicate the upper and lower quartiles, with a line at the median. Outliers are shown as circles and identified via the interquantile range rule. **b** Optimisation of the number of imaging clusters.

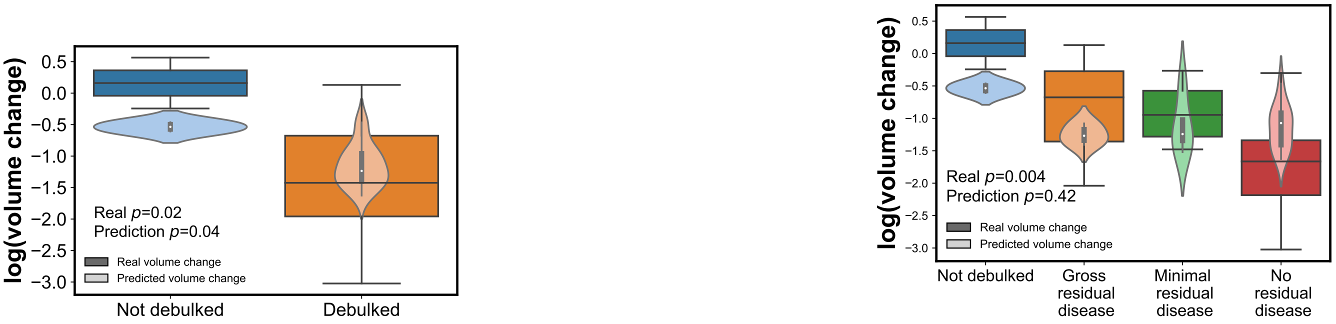

(a) Binary response

(b) Gradual response

Supplementary Fig. 5. Relative change in total volume predicted by the model (violin plot, pastel colour) and observed in the data (box plot, darker colour) versus surgical debulking status computed for the hold-out validation cohort (n=20). p values are computed using the Mann-Whitney U test **a** and Spearman correlation **b**, respectively, both two-sided. Boxes indicate the upper and lower quartiles, with a line at the median. Outliers are shown as circles and identified via the interquantile range rule.

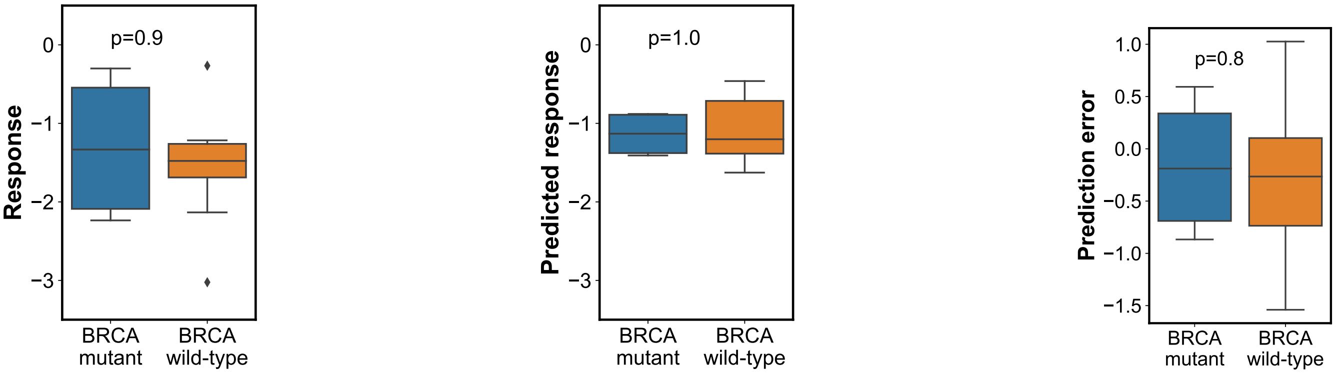

(a) Observed response

(b) Predicted response

(c) Prediction error

Supplementary Fig. 6. Effect of *BRCA1/2* mutation status in the hold-out validation cohort for which *BRCA1/2* mutation status was available (n=15) on **a** observed response; **b** predicted response; and **c** the prediction error, calculated as the difference between the responses. p values are computed using the Mann-Whitney U test (two-sided). Boxes indicate the upper and lower quartiles, with a line at the median. Outliers are shown as circles and identified via the interquantile range rule.

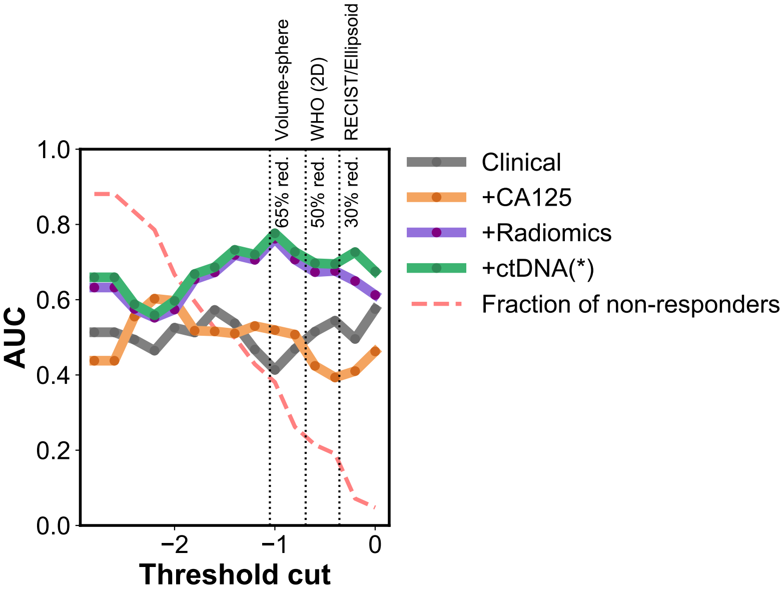

Supplementary Fig. 7. Performance of the model as a binary classifier applied to the external validation cohort, using a dichotomised version of the volumetric response as a reference. The vertical axis shows the area under the receiver-operating characteristic curve, and the horizotal axis shows the threshold applied on the observed volumetric response to create the two response categories. The different colours correspond to the different models evaluated. The red dashed line corresponds to the fraction of non-responders obtained after applying the different thresholds. The vertical dotted lines indicate the thresholds corresponding to different response criteria.(*) ctDNA values for the full integrated model were imputed using the corresponding training set averages.

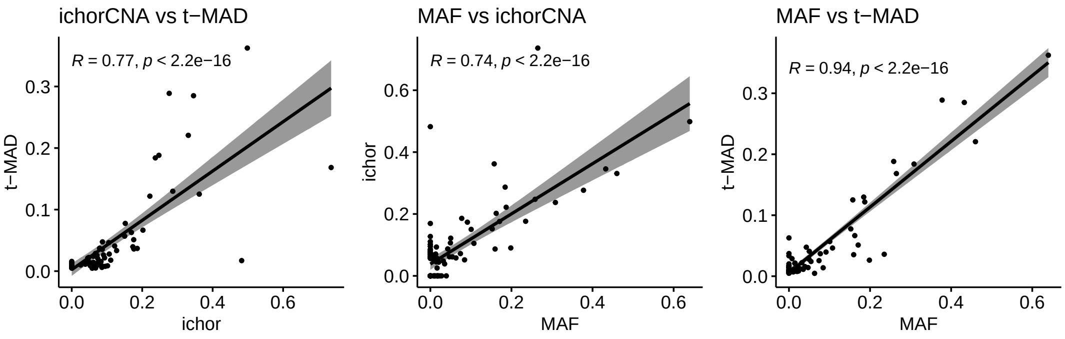

Supplementary Fig. 8. We used ichorCNA, a tool for estimating the fraction of tumour in cell-free DNA from ultra-low-pass whole genome sequencing, to explore differences with respect to our chosen biomarkers. ichorCNA is highly correlated (Pearson R) with MAF **a** and t-MAD **b**, which are also correlated between them **c**. We calculated ichorCNA values according to published methodology (1). These figures include both the training set and the hold-out validation set. The grey band indicates the 95% confidence interval around the regression line. *p* values were calculated using the Pearson correlation coefficient (two-sided).

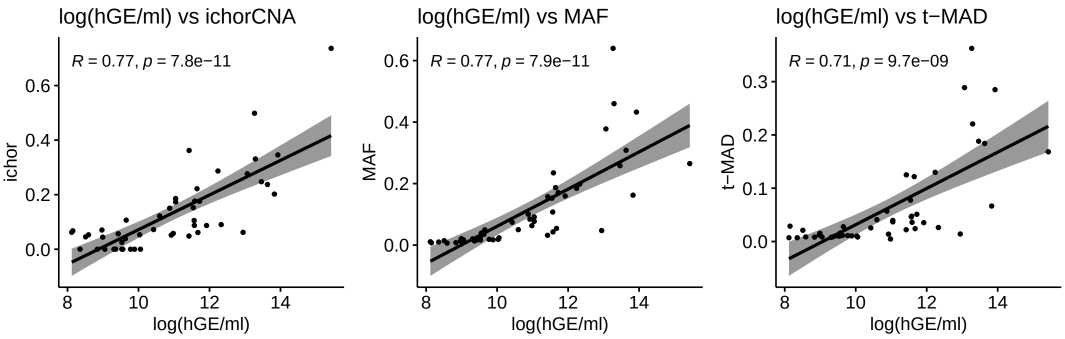

Supplementary Fig. 9. We computed haploid genome equivalents per millilitre (hGE/ml), to explore differences with respect to our chosen biomarkers. We found that hGE/ml is highly correlated (Pearson R) with ichorCNA **a**, MAF **b** and t-MAD **c**. We computed hGE/ml according to previously described methodology (2). We also calculated ichorCNA values according to published methodology (1). These figures include both the training set and the hold-out validation set. The grey band indicates the 95% confidence interval around the regression line. *p* values were calculated using the Pearson correlation coefficient (two-sided).

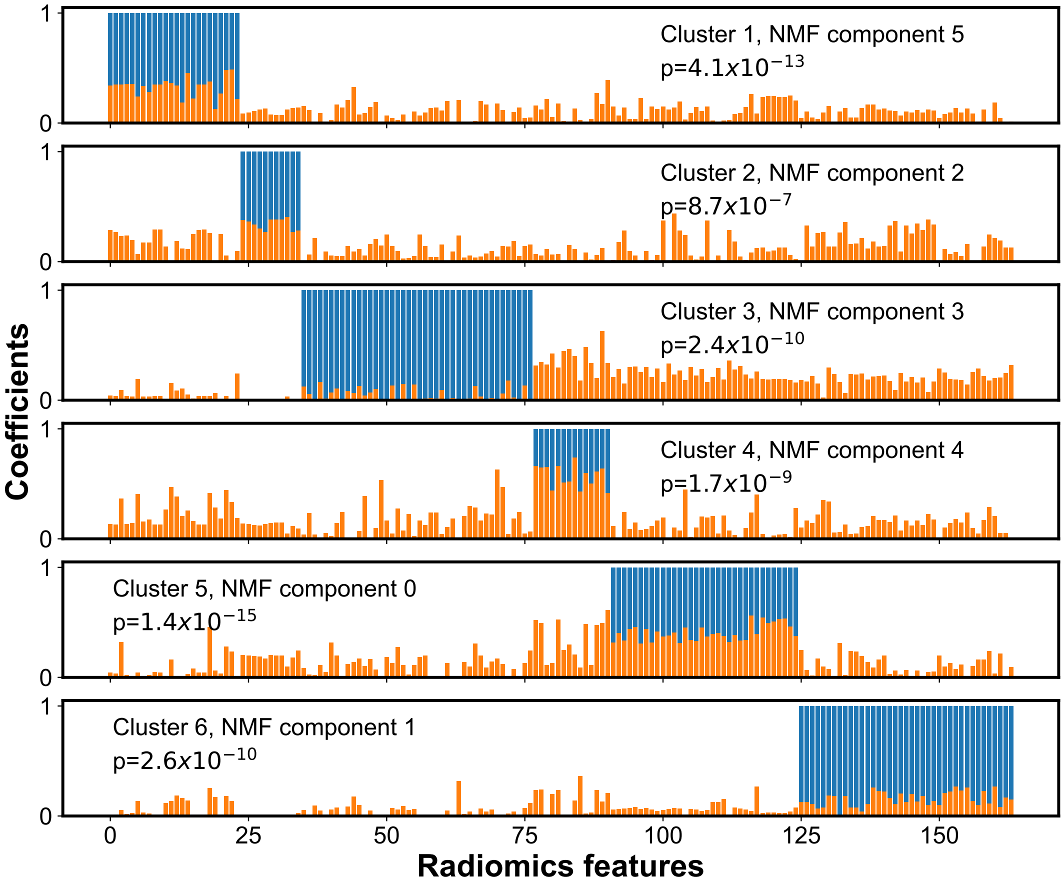

Supplementary Fig. 10. To explore the robustness of the clusters identified we implemented non-negative matrix factorization as an alternative clustering method. We matched the number of clusters to 6, used a coordinate descent solver and an initialization based on non-negative random matrices, following the scikit-learn implementation. We found that each the resulting 6 clusters was highly concordant with one of the corresponding hierarchical clusters (Mann-Whitney U test $p < 10^{-7}$ in all cases, two-sided), with the exception of cluster 3, the 'unassigned' cluster.

B. Supplementary tables

Table 1. Breakdown of the age, stage, performance status, response status, and treatment regimens received by patients in the different cohorts.

	Training set	Hold-out set	External validation set	Total
Age at diagnosis	64.4 (29-90)	63.9 (47-83)	63.1 (35-85)	63.9 (29-90)
Stage				
3B	0	1	0	1
3C	42	16	31	89
4A	13	0	11	24
4B	17	3	0	20
Performance status				
Not recorded	8	1	42	51
0	33	10	0	43
1	22	8	0	30
2	9	1	0	10
RECIST 1.1				
Response	36	12	30	78
Stable	35	7	12	54
Progression	1	1	0	2
Combination therapy	52	16	39	107
Weekly Carboplatin and Paclitaxel	2	0	0	2
3-weekly Carboplatin and Paclitaxel	33	9	39	81
Weekly Carboplatin, 3-weekly Paclitaxel	13	5	0	18
Carboplatin monotherapy	17	3	3	23
Doxorubicin	3	1	0	4

Table 2. Slice thickness of the CT scans in the training and validation datasets.

Dataset	Slice spacing [cm] (number of scans)
Training set	0.069 (1), 0.15 (1) , 0.25 (1), 0.3 (2), 0.375 (19), 0.5 (48)
Hold-out validation set	0.3 (1), 0.375 (4), 0.5 (15)
External validation set	0.069 (1), 0.2 (12), 0.41 (2), 0.5 (31)

Table 3. Mapping between FIGO stages and ordinal numbers used for predictive modelling.

FIGO stage	Ordinal
1A	1
1B	2
1C	3
2A	4
2B	5
3A	6
3B	7
3C	8
4A	9
4B	10

Table 6. Performance results for the cross-validation training set and the two validation sets. *p* values are two-sided.

	Training set (CV)	Hold-out set			External set		
Model	MSE change (%)	MSE change (%)	Spearman r	Pearson r	MSE change (%)	Spearman r	Pearson r
Clinical	–	–	r_S=0.49, p=0.03	r_P=0.30, p=0.20	–	r_S=-0.00, p=0.98	r_P=0.03, p=0.85
+ CA-125	-5.2	-1.5	r_S=0.37, p=0.11	r_P=0.32, p=0.17	+0.7	r_S=0.04, p=0.80	r_P=0.03, p=0.84
+ Radiomics	-24.0	-15.4	r_S=0.50, p=0.02	r_P=0.50, p=0.02	-8.6	r_S=0.32, p=0.04	r_P=0.29, p=0.07
+ ctDNA	-25.0	-14.0	r_S=0.50, p=0.02	r_P=0.49, p=0.03	-10.8(*)	r_S=0.36, p=0.02(*)	r_P=0.32, p=0.04(*)

(*) ctDNA values in the external validation cohort were imputed using training set averages.

Table 4. Hyperparameter ranges used in the cross-validation model optimisation.

Algorithm	Hyperparameter	Range
Elastic net	Penalty coefficient α	$10^{-8} - 10$ (200 log steps)
Elastic net	L1 ratio	0.1–1.0 (steps of 0.1)
SVR	Kernel coefficient γ	$10^{-9} - 10^{3}$ (100 log steps)
SVR	Regularisation parameter C	$10^{-3} - 10^{3}$ (100 log steps)
RF	Max. depth	3 or automatic
RF	Num. of estimators	5, 10, 25, 50, 100
RF	Max. features in split	0.05, 0.1, 0.2, 0.5, 0.7
RF	Min. samples in split	2, 3, 6, 10, 12, 15

Table 5. Optimised hyperparameters used in each of the algorithms and seeds.

Algorithm	Parameter	Seed 1	Seed 2	Seed 3	Seed 4	Seed 5
Elastic net	Penalty coefficient α	0.049	0.542	0.667	0.667	0.667
Elastic net	L1 ratio	0.200	0.400	0.300	0.200	0.300
SVR	Regularisation parameter C	869.749	2.154	10.000	869.749	657.933
SVR	Kernel coefficient γ	0.011	0.033	0.011	0.011	0.000
RF	Max. depth	Automatic	Automatic	3.0	3.0	3.0
RF	Max. features in split	0.7	0.5	0.1	0.1	0.1
RF	Min. samples in split	12.0	2.0	10.0	6.0	10.0
RF	Num. estimators	10.0	50.0	5.0	5.0	10.0

Table 7. Key descriptors of the response variable.

Dataset	Min	Max	Mean	Median	Standard Deviation
Training	-3.492	0.598	-1.218	-1.197	0.906
Hold-out	-3.023	0.565	-1.216	-1.338	0.912
External	-3.649	0.091	-1.497	-1.397	1.003

Table 8. Performance (Spearman r) of the full integrated model after removing volume-related individual features and clusters of features during inference. *p* values are two-sided.

Features dropped	Performance
None	r_S=0.50, p=0.02
Mean volume	r_S=0.46, p=0.04
Vol. infrarenal LNs	r_S=0.48, p=0.03
Cluster 1	r_S=0.58, p=0.007
Cluster 4	r_S=0.55, p=0.01

Table 9. List of all non-imaging features used in the predictive models, and their means, medians, standard deviations and ranges for the training, hold-out validation and external validation sets.

		Training set				Hold-out validation set				External validation set			
Class	Feature name	Mean	Median	σ	Range	Mean	Median	σ	Range	Mean	Median	σ	Range
Clinical	Stage	8.65	8.0	0.84	8-10	8.25	8.0	0.77	7-10	8.26	8.0	0.44	8-9
Clinical	Age at diagnosis	64.43	66.5	12.19	29.0-90.0	63.85	66.0	10.3	47.0-83.0	63.14	65.0	12.19	35-85
Clinical	Num. sessions before 2nd CT	4.056	3.0	1.95	2.0-9.0	4.35	3.0	2.17	3.0-9.0	3.4	3.0	0.73	3-6
Clinical	Num. days between 1st CT and treatment start	25.0	26.0	13.2	1.0-68.0	21.55	23.5	15.61	1.0-70.0	33.048	27.0	21.94	0.0-99.0
Clinical	Num. days between 1st and 2nd CTs	80.97	80.5	18.59	48.0-156.0	76.15	75.5	19.27	48.0-143.0	84.38	78.0	27.45	45.0-173.0
Clinical	Received Doxorubicin	0.042	0.0	0.2	0.0-1.0	0.05	0.0	0.22	0.0-1.0	0.0	0.0	0.0	0.0-0.0
Clinical	Paclit. and Carbop. weekly	0.028	0.0	0.16	0.0-1.0	0.0	0.0	0.0	0.0-0.0	0.0	0.0	0.0	0.0-0.0
Clinical	Paclit. and Carbop. 3-weekly	0.46	0.0	0.5	0.0-1.0	0.45	0.0	0.5	0.0-1.0	0.93	1.0	0.26	0.0-1.0
Clinical	Paclit. weekly, Carboplat. 3-weekly	0.18	0.0	0.38	0.0-1.0	0.25	0.0	0.43	0.0-1.0	0.0	0.0	0.0	0.0-0.0
Clinical	Only Carboplatin	0.24	0.0	0.42	0.0-1.0	0.15	0.0	0.36	0.0-1.0	0.071	0.0	0.26	0.0-1.0
Clinical	Received Paclitaxel	0.72	1.0	0.45	0.0-1.0	0.8	1.0	0.4	0.0-1.0	0.93	1.0	0.26	0.0-1.0
CA-125	Baseline CA-125	2.1e+03	972.0	2.6e+03	11-1.2e+04	729.15	523.0	637.69	33-2.1e+03	2.3e+03	899.5	5.1e+03	74-3.1e+04
ctDNA	ctDNA tMAD	0.044	0.012	0.073	0.0047-0.36	0.014	0.0087	0.013	0.005-0.057	–	–	–	–
ctDNA	ctDNA TP53 MAF	0.071	0.012	0.13	0.0-0.64	0.014	0.0	0.025	0.0-0.1	–	–	–	–
ctDNA	ctDNA TP53 mutation status	0.57	1.0	0.5	0.0-1.0	0.4	0.0	0.49	0.0-1.0	–	–	–	–

References

1. VA Adalsteinsson, et al., Scalable whole-exome sequencing of cell-free dna reveals high concordance with metastatic tumors. *Nat. Commun.* **8**, 1–13 (2017).
2. F Scherer, et al., Distinct biological subtypes and patterns of genome evolution in lymphoma revealed by circulating tumor dna. *Sci. Transl. Medicine* **8** (2016).
