# Supplementary material for: Integrated radiogenomics models predict response to neoadjuvant chemotherapy in high grade serous ovarian cancer
Source: Nat Commun. 2023 Oct 24;14:6756. doi: 10.1038/s41467-023-41820-7 (PMC10598212; doi:10.1038/s41467-023-41820-7)
Supplement: Supplementary file 2 — Description of Additional Supplementary Files [file 41467_2023_41820_MOESM2_ESM.docx]

**Description of Additional Supplementary Files**

Supplementary Data 1

Description: Contains one table with the mean, median, standard deviation and range of all the features used in the analysis in the training, hold-out, and external validation datasets.

Supplementary Movie 1

Description:

Supplementary Software 1

Description:
